# Radiographic and clinical outcomes following MIS-TLIF in patients with adult lumbar degenerative scoliosis

**DOI:** 10.1186/s13018-018-0764-7

**Published:** 2018-04-19

**Authors:** Yongfei Zhao, Yan Liang, Keya Mao

**Affiliations:** 1 The General Hospital of Chinese People’s Liberation Army (301 hospital), Beijing, 100853 China; 2 Peking University People’s Hospital, Beijing, 100044, 314 China

**Keywords:** MIS-TLIF, Adult lumbar degenerative scoliosis, Cobb angle, Visual analog scale, Oswestry disability index

## Abstract

**Background:**

Patients suffering from adult lumbar degenerative scoliosis (ALDS) are commonly complicated with advanced age, osteoporosis, cardiopulmonary insufficiency, and some other medical comorbidity. Therefore, the traditional open surgery can lead to high rate of postoperative complications. The purposes of this study were to introduce our experiences and explore the efficacy and feasibility of minimally invasive transforaminal lumbar interbody fusion (MIS-TLIF) in the treatment of patients with ALDS.

**Methods:**

From January 2008 to January 2014, a retrospective study of 22 patients with ALDS treated with MIS-TLIF was followed up at least 2 years. All patients suffered from one-level lumbar stenosis, and the nerve root block was performed to make sure the exact level. The clinical and radiographic outcomes were evaluated preoperatively and at the time of 2-year follow-up.

**Results:**

The mean visual analog scale (VAS) back pain scores decreased from 6.2 ± 1.8 preoperatively to 2.2 ± 0.7 at 2-year follow-up (*P* < 0.05), and the mean VAS leg pain scores decreased from 8.2 ± 0.7 preoperatively to 1.4 ± 1.4 at 2-year follow-up (*P* < 0.05). The Oswestry Disability Index score improved from 62.4 ± 16.1% preoperatively to 24.2 ± 9.3% at 2-year follow-up (*P* < 0.05). The average lumbar curve was 20.7° ± 7.0° preoperatively and 12.7° ± 7.1° at 2-year follow-up (*P* < 0.05). The lumbar lordosis changed from − 39.5° ± 13.6° to − 43.6° ± 10.6° at 2-year follow-up (*P* < 0.05). Solid fusion was achieved in all patients.

**Conclusion:**

The technique of MIS-TLIF can be used to treat the patients with ALDS whose symptom is mainly from one-level lumbar stenosis, achieving favorable clinical outcomes and good fusion, with less blood loss and complications.

## Background

The adult lumbar degenerative scoliosis (ALDS), described as “de novo” scoliosis, was defined as a curve > 10° due to degeneration of the facets and discs [1]. The patients of ALDS usually suffer from radicular or neurogenic claudication symptoms and back pain which make the surgery necessary [2–5]. However, the patients of ALDS are usually complicated with advanced age, osteoporosis, cardiopulmonary insufficiency, and other medical comorbidities, which contribute to the high rate of postoperative complications. Traditional open surgery has been associated with a major complication rate as high as 28–86% [6–8], and the risks of morbidity have been shown to increase with advancing age [9].

In order to lower the incidence of the complication, several minimally invasive methods of treatment for ALDS have been advocated [10–12]. However, the best option for this disorder is still controversial. The purposes of this study are to introduce our experience and explore the efficacy and feasibility of the technique of minimally invasive transforaminal lumbar interbody fusion (MIS-TLIF) for patients of ALDS whose symptom is mainly single-level radicular pain or neurogenic claudication, without dynamic back pain.

## Methods

### Patients

From January 2008 to January 2014, 22 consecutive patients with ALDS, treated with MIS-TLIF in our hospital, were retrospectively analyzed. They were followed up at least 2 years. There were 8 males and 14 females, with an average age of 63.7 years (range 47–79 years). Inclusion criteria were (1) Cobb angle above 10°, (2) posterior-only procedure for adult scoliosis correction, (3) suffered from one-level lumbar stenosis and the nerve root block was performed to make sure the exact level, (4) treated with MIS-TLIF technique, (4) availability of radiographic examinations (full-length AP and lateral radiographs) and clinical data (inpatient medical records and questionnaire), (5) participated in non-operative therapies, including bracing, resting, physiotherapy, and analgesics, without adequate relief of their symptoms. Exclusion criteria were (1) idiopathic curves; (2) prior lumbar fusion surgery; (3) other comorbidities, such as neoplasia, trauma, and infection; (4) patients whose symptoms are mainly dynamic or fatigue back pain.

### Study measures

Study measures were obtained through a review of inpatient medical records and questionnaire. The primary measures of this study were blood loss, surgery time, the time to ambulation, postoperative hospital stay, visual analog score (VAS), and Oswestry Disability Index (ODI).

### Radiologic assessment

Radiographic examinations were performed preoperatively, postoperatively, and at the time of every follow-up. Radiographic data were collected and evaluated preoperatively and at the time of 2-year follow-up. The Cobb angle of the lumbar curve was measured using the standard Cobb’s method on an anteroposterior radiograph, and the lumbar and pelvic parameters were measured on a lateral radiograph including lumbar lordosis (LL), sacrum slope (SS), and pelvic tilt (PT). The radiologic films and CT taken at 2-year follow-up were utilized to assess fusion. The fusion criteria were based on Bridwell interbody fusion grading system (Table 1), and the assessments were performed by two independent assessors.

**Table 1:** Bridwell interbody fusion grading system

Grade	Description
I	Fused with remodeling and trabeculae are present
II	Graft intact, not fully remodeled and incorporated, but no lucency is present
III	Graft intact, potential lucency is present at the top and bottom of the graft
IV	Fusion is absent with collapse/resorption of the graft

### Surgical procedures

All cases were treated with nerve root block to make sure the level where the pain comes from. The nerve root block was performed under the C-arm fluoroscopy. The puncture needle entered the foramen intervertebrale in a proper entry point and angle. And the lidocaine was used to block the nerve root. Then, the MIS-TLIF surgery was performed.

**Fig. 1:**
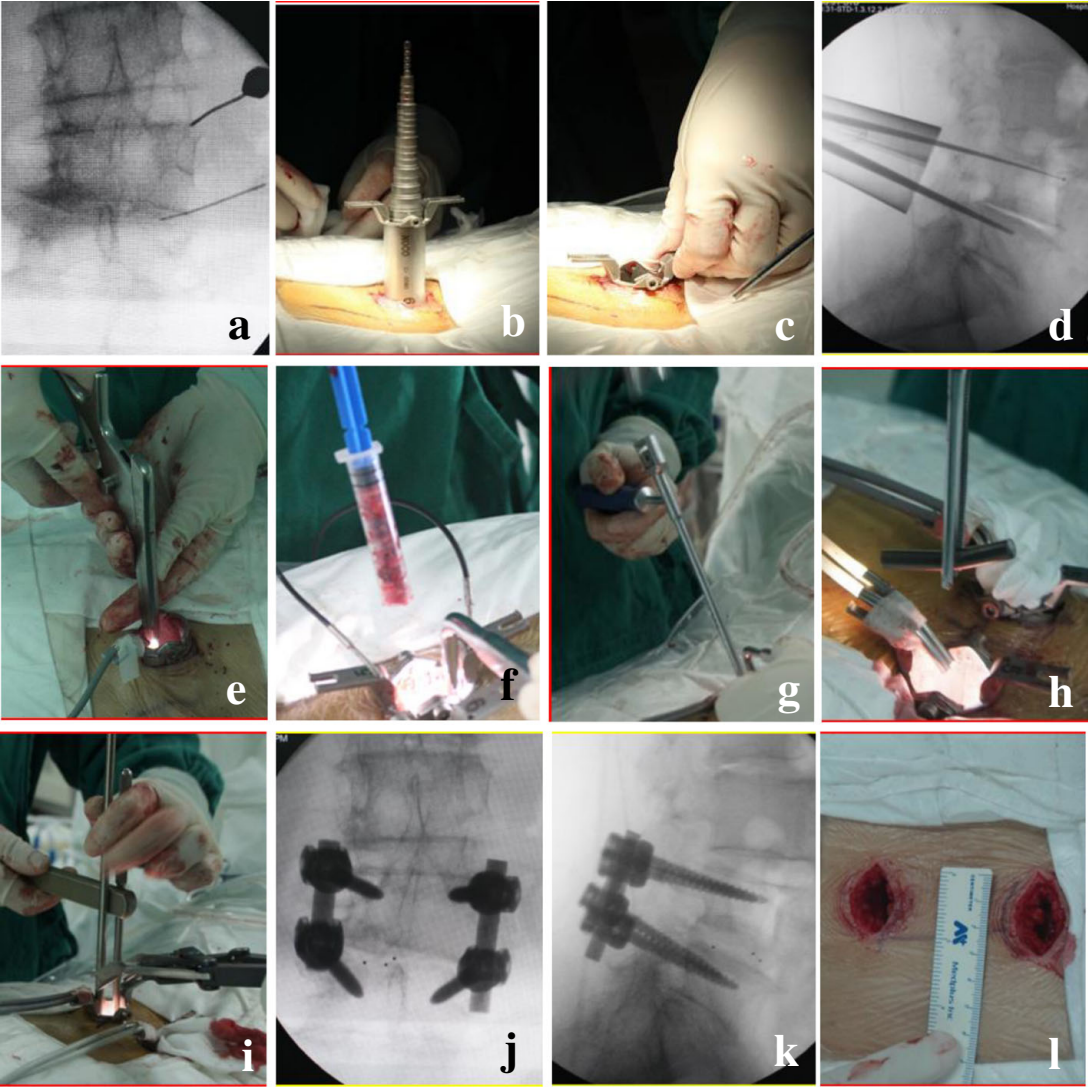
The MIS-TLIF technique. **a** Position the level under the C-arm fluoroscopy. **b**, **c** The tubular retractor was placed. **d** The road of pedicle screw was prepared. **e** The progress of decompression was performed. **f**, **g** Bone and cage interbody graft was inserted. **h**, **i**: The progress of compression was performed. **j**, **k** The postoperative X-ray showed good result. **l** The incision was about 2.5 cm

Under general anesthesia, the patient was placed in a prone position on the operating table. The needle is used to position the level under the C-arm fluoroscopy. A 2.5-cm incision away from the center line 2 cm was made, and a tubular retractor was placed in a proper angle. Then, the trajectory of pedicle screw was prepared bare-handed and sealed by bone wax. The isthmus, the posterior arch of the vertebrae, the inferior joint facet, and the ligamentum flavum were resected. These local bones were kept for autograft during the interbody fusion. The nerve root was identified, and the canal of nerve root was clearly decompressed. Then, discectomy and endplate preparation were performed, and the disc space was packed with the autograft bones. A cage interbody graft was then inserted and commonly placed relatively to the concave side to restore lumbar lordosis and decrease lumbar curve. Another 2.5-cm incision was made on the contralateral side, and the same procedure of instrumentation and decompression was performed. The lumbar pedicle screws were inserted bilaterally, and the progress of compression on the convex side and distraction on the concave side was performed. Finally, the incision was sewn up without drainage (Fig. 1).

### Statistical analysis

Data were expressed as mean ± standard deviations for variables. Preoperative and postoperative differences were performed using a paired *t* test, and statistical significance was set at *P* < 0.05. All analyses were carried out using the SPSS (Statistical Package for the Social Sciences) version 17.

## Results

### Surgical results

The average surgical time was 153.3 ± 26.3 min (range 105–220 min) with a mean intraoperative blood loss of 175.0 ± 83.4 ml (range 50–325 ml). The hospital stay was 5.4 ± 0.9 days (range 4–7 days). The time to ambulation was 2.5 ± 0.9 days (range 1–4 days) postoperatively (Table 2).

**Table 2:** Patient demographics and operative data

Variables	Data
Age (years)	63.7
Sex	
Male	8
Female	14
Level	
L3/L4	3
L4/L5	11
L5/S1	8
Surgery time (min)	153.3 ± 26.3
Blood loss (ml)	175.0 ± 83.4
Time to ambulation (days)	2.5 ± 0.9
Hospital stay (days)	5.4 ± 0.9

### Clinical results

The mean VAS back pain scores decreased from 6.2 ± 1.8 preoperatively to 2.2 ± 0.7 at 2-year follow-up (*P* < 0.05), and the mean VAS leg pain scores decreased from 8.2 ± 0.7 preoperatively to 1.4 ± 1.4 at 2-year follow-up (*P* < 0.05). The ODI score improved from 62.4 ± 16.1% preoperatively to 24.2 ± 9.3% at 2-year follow-up (*P* < 0.05). All patients were satisfied with the surgical results (Table 3).

**Table 3:** Radiographic and clinical outcomes in 16 patients

Variables	Preoperative	2-year postoperative follow-up	*t*	*P*
Cobb	20.7° ± 7.0°	12.7° ± 7.1°	8.5	0.000 < 0.05
LL	− 39.5° ± 13.6°	− 43.6° ± 10.6°	3.5	0.014 < 0.05
SS	28.5° ± 9.1°	33.5° ± 6.1°	− 3.8	0.006 < 0.05
PT	20.2° ± 5.5°	14.9° ± 6.4°	3.5	0.019 < 0.05
VAS(back)	6.2 ± 1.8	2.2 ± 0.7	6.2	0.000 < 0.05
VAS(leg)	8.2 ± 0.7	1.4 ± 1.4	12.9	0.000 < 0.05
ODI(%)	62.4 ± 16.1	24.2 ± 9.3	8.1	0.000 < 0.05

### Radiological results

**Fig. 2:**
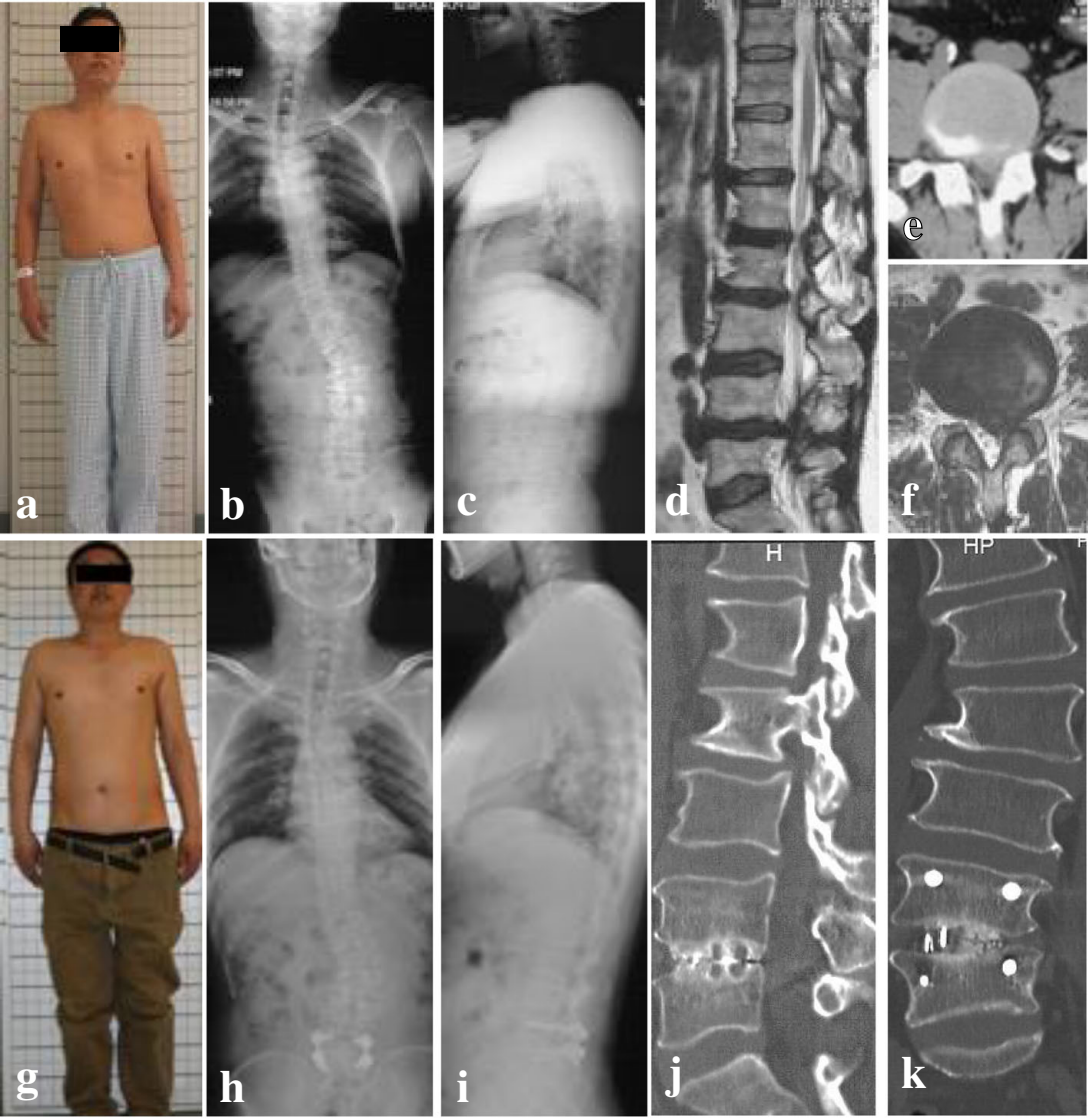
A 65-year-old male patient suffering from adult lumbar degenerative scoliosis. The main complains were severe back and left leg pain complicated with intermittent claudication. **a** Preoperative view photograph showed that the trunk tilt to the left is obvious. **b**, **c** Preoperative X-ray showed the Cobb angle was 32°, and the coronal was imbalanced. **d**, **e**, **f** Preoperative CT and MRI showed the L4/5 disc herniation. **g** The view photograph of the 2-year follow-up showed that the trunk tilt is not obvious. **h**, **i** The X-ray of the 2-year follow-up showed the Cobb angle was 23°, and the coronal was balanced. **j**, **k** The CT of the 2-year follow-up showed grade 1 fusion

The levels of surgery were L3/4 in 3 cases (13.6%), L4/5 in 11 cases (50%), and L5/S1 in 8 cases (36.4%). The mean Cobb angle decreased from 20.7° ± 7.0° preoperatively to 12.7° ± 7.1° at 2-year follow-up with a mean correction of 8° (*P* < 0.05). The lumbar lordosis changed from − 39.5° ± 13.6° preoperatively to − 43.6° ± 10.6° at 2-year follow-up (*P* < 0.05). The pelvic tilt decreased from 20.2° ± 5.5° preoperatively 14.9° ± 6.4° to at 2-year follow-up (*P* < 0.05). The sacrum slope changed from 28.5° ± 9.1° preoperatively to 33.5° ± 6.1° at 2-year follow-up (*P* < 0.05). All patients achieved grade 1 fusion at the final follow-up according to radiological evidence, and no obvious loss of correction occurred (Table 3) (Fig. 2).

### Complication

There was one dura tear with cerebrospinal fluid leakage, which was repaired during operation. One patient suffered from pneumonia and recovered after antibiotic treatment. One patient developed the adjacent segment degeneration which will need another surgery. There was no complication of neurologic injury, wound infection, or non-union. There had been no breakage or failure of any screw or rod.

## Discussion

The ALDS is usually caused by the degeneration of the spine. The prevalence of ALDS is reported to be 6% [13–16]. The treatment of the ALDS is in an ongoing debate [17–19]. In order to choose the best option, the Lenke-Silva [1] classification is described to instruct the treatment. The open surgery to correct deformity, which can get good coronal and sagittal balance, is becoming popular. However, for the patients with ALDS, the average age is in the 60s [20]. The advanced age of the patient is often complicated with medical comorbidities, which will add additional challenges to the surgery and increase the complication rate of the patient. According to the literature, the traditional open surgery has been associated with a major complication rate as high as 28–86% [21], and the risks of morbidity have been shown to increase with advancing age [22].

To combat these challenges, the minimally invasive surgeries have been developed for the treatment of ALDS [23–25]. The minimally invasive spinal surgery can reduce intraoperative blood loss, lower infection rates, and quicker mobilization, which will be highly desired in the adult lumbar degenerative scoliosis population [26]. Rosen’s study [27] proved that patients older than 75 with significant medical comorbidities underwent minimally invasive spinal surgery for spinal canal decompression could be efficient and safe. The minimally invasive surgical treatment of ALDS is increasingly being recognized as safe and effective.

The technique of MIS-TLIF was first described by Foley [28], using tubular retractors under radiological guidance by a muscle-dilating approach, which can reduce the amount of iatrogenic muscle and soft tissue injuries, which was confirmed by many other surgeons [29–31]. Lee [32]found that the MIS-TLIF surgery generally has minimal blood loss compared with the open surgery, and the patients treated by open surgery generally take three times as long to start walking, and they stay twice as long in the hospital.

ALDS patients typically present with symptoms of low back pain, lower back fatigue, neurogenic claudication, lumbosacral radicular pain, and a progressive deformity. The golden standard treatments are decompression, fusion, and deformity correction [17, 23]. However, the patients with ALDS have different clinical manifestations, which need various surgical principles and techniques. Therefore, we advocated the precise treatment for these patients, to solve the symptom rather than restore the alignment. The surgery should be as minor as possible to reduce the complications. The nerve root block is a necessary procedure for precise treatment, which can make sure the level where the pain comes from. In our study, 22 patients with ALDS whose symptom is mainly single-level radicular pain or neurogenic claudication were treated with the technique of MIS-TLIF and achieve a good decompression, instrumentation, and fusion with less injury, relieving patients’ pain, and gaining satisfactory clinical outcome eventually. From the data, for the patients with ALDS who suffered from one-level lumbar stenosis, the technique of MIS-TLIF can be accomplished within shorter operative time, to be associated with much less blood loss and shorter hospital stays [1, 13], which result in considerably less patient morbidity, less cost, and earlier rehabilitation.

Besides, the deformity correction is another important consideration. In our study, the mean Cobb angle decreased from 20.7° ± 7.0° to 12.7° ± 7.1° with a mean correction of 8°. And the lumbar lordosis changed from − 39.5° ± 13.6° to − 43.6° ± 10.6°. With a thorough decompression, disc removement, interbody bone graft and cage instrument, compression on the convex side and distraction on the concave side, and the local deformity could be corrected to some degree. During the surgery, we commonly use a large cage placed relatively to the concave side to restore lumbar lordosis and decrease lumbar curve. Besides, the painful stimulus caused by disc herniation or stenosis is removed; the nerve root, muscle, and ligament are relaxed which are helpful for deformity correction. The change of the parameters demonstrated that the technique of MIS-TLIF could improve the balance of the patients to some degree. Earlier studies have shown a significant positive correlation between the radiographic results and clinical outcomes in the surgical treatment of ALDS, which is in accordance with our study [14, 33]. But the deformity of patients in our study is still there, and the outcome of deformity correction is not satisfied. Therefore, the long-term evaluation should be future studied.

There are limitations in this study. Firstly, due to the steep learning curve of MIS-TLIF and the characteristic of the ALDS patients, it may be difficult for junior surgeons to do the procedure, which could result in increased surgical time and blood loss and more complications such as dura tear and nerve root injury. All the procedures in this study were accomplished by the corresponding author, an experienced senior surgeon of MIS-TLIF. Secondly, the patients with ALDS present many different symptoms. Right now, the technique of MIS-TLIF is only suitable for patients whose symptom is mainly single-level radicular pain or neurogenic claudication. The indication of MIS-TLIF for ALDS is relatively narrow. Besides, the number of the cases in our study is relatively small, and the time of follow-up is relatively short, so the larger cases of long-term observation research should be future studied.

## Conclusion

In conclusion, for the patients with ALDS, the deformity correction is not that necessary for some patients; we advocated the precise treatment to relieve the main pain and improve the symptom. For the patients with ALDS who suffered from one-level lumbar stenosis, the technique of MIS-TLIF was safe and effective. The technique of MIS-TLIF is generally associated with less blood loss and pain, earlier ambulation and discharge from hospital, and reduced incidence of the complications. Although the technique is not suitable for all patients with ALDS, it may be a suitable option for some patients.

## Abbreviations

ALDS: Adult lumbar degenerative scoliosis
LL: Lumbar lordosis
ODI: Oswestry Disability Index
PT: Pelvic tilt
SS: Sacrum slope
VAS: Visual analog score
